# Supplementary material for: A human antibody reveals a conserved site on beta-coronavirus spike proteins and confers protection against SARS-CoV-2 infection
Source: Sci Transl Med. 2022 Feb 8:eabi9215. doi: 10.1126/scitranslmed.abi9215 (PMC8939767; doi:10.1126/scitranslmed.abi9215)
Supplement: Supplementary file 1 — Figs. S1 to S11 Tables S1 and S2 References (93–98) [file scitranslmed.abi9215_sm.pdf]

Science Translational Medicine
AAAS

Supplementary Materials for

A human antibody reveals a conserved site on beta-coronavirus spike proteins and confers protection against SARS-CoV-2 infection

Panpan Zhou *et al.*

Corresponding authors: Thomas F. Rogers, trogers@scripps.edu; Ian A. Wilson, wilson@scripps.edu; Dennis R. Burton, burton@scripps.edu; Raiees Andrabi, andrabi@scripps.edu

DOI: 10.1126/scitranslmed.abi9215

The PDF file includes:

Figs. S1 to S11
Tables S1 and S2
References (*93–98*)

Other Supplementary Material for this manuscript includes the following:

MDAR Reproducibility Checklist
Data file S1

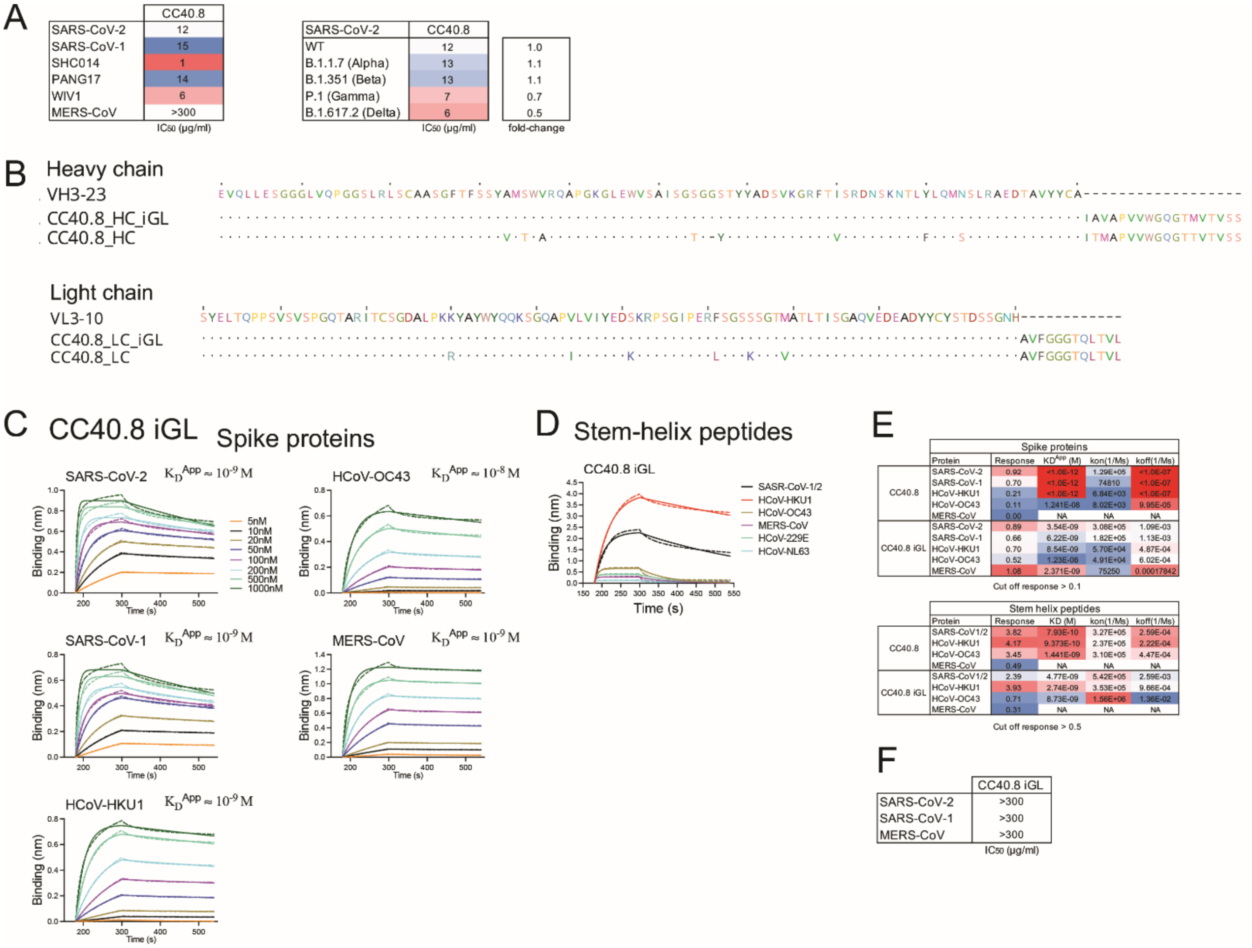

Fig. S1. CC40.8 mature and CC40.8 iGL antibodies bind to spike proteins and stem-helix peptides and mature antibody neutralizes pseudotyped coronaviruses.

(A) IC_{50} neutralization of CC40.8 broadly neutralizing antibody (bnAb) is shown for sarbecoviruses (SARS-CoV-2, SARS-CoV-1, SHC014, Pang17 and WIV1), MERS-CoV and SARS-CoV-2 variants of concern (alpha (B.1.1.7), beta (B.1.351), gamma (P.1) and delta (B.1.617.2)).

(B) Sequence alignment of CC40.8 heavy and light chains with their corresponding germline V-gene sequences (VH3-23 and VL3-10) is shown with the design of CC40.8 antibody inferred germline (iGL) gene sequences. Dots represent identical residues and dashes represent gaps introduced to preserve the alignment.

(C) BioLayer Interferometry (BLI) binding is shown for CC40.8 iGL antibody with human β-HCoV soluble spike proteins. Apparent binding constants (K_D^{App}) for each antibody-antigen interaction are indicated. The raw experimental curves are shown as dashed lines, and the solid lines are the fits.

(D) BLI binding is shown for CC40.8 iGL antibody (Ab) to 25-mer stem peptides derived from all HCoV spike proteins. The kinetic curves are fit with a 1:1 binding mode.

(E) Binding kinetics (K_D^{App} (spike proteins), K_D (stem-helix peptides) k_{on} and k_{off} constants) of CC40.8 and CC40.8 iGL antibodies with human β-HCoV soluble spike proteins and the 25-mer β-HCoV stem peptides are shown.

(F) IC_{50} neutralization of CC40.8 iGL is shown for sarbecoviruses (SARS-CoV-2 and SARS-CoV-1) and MERS-CoV.

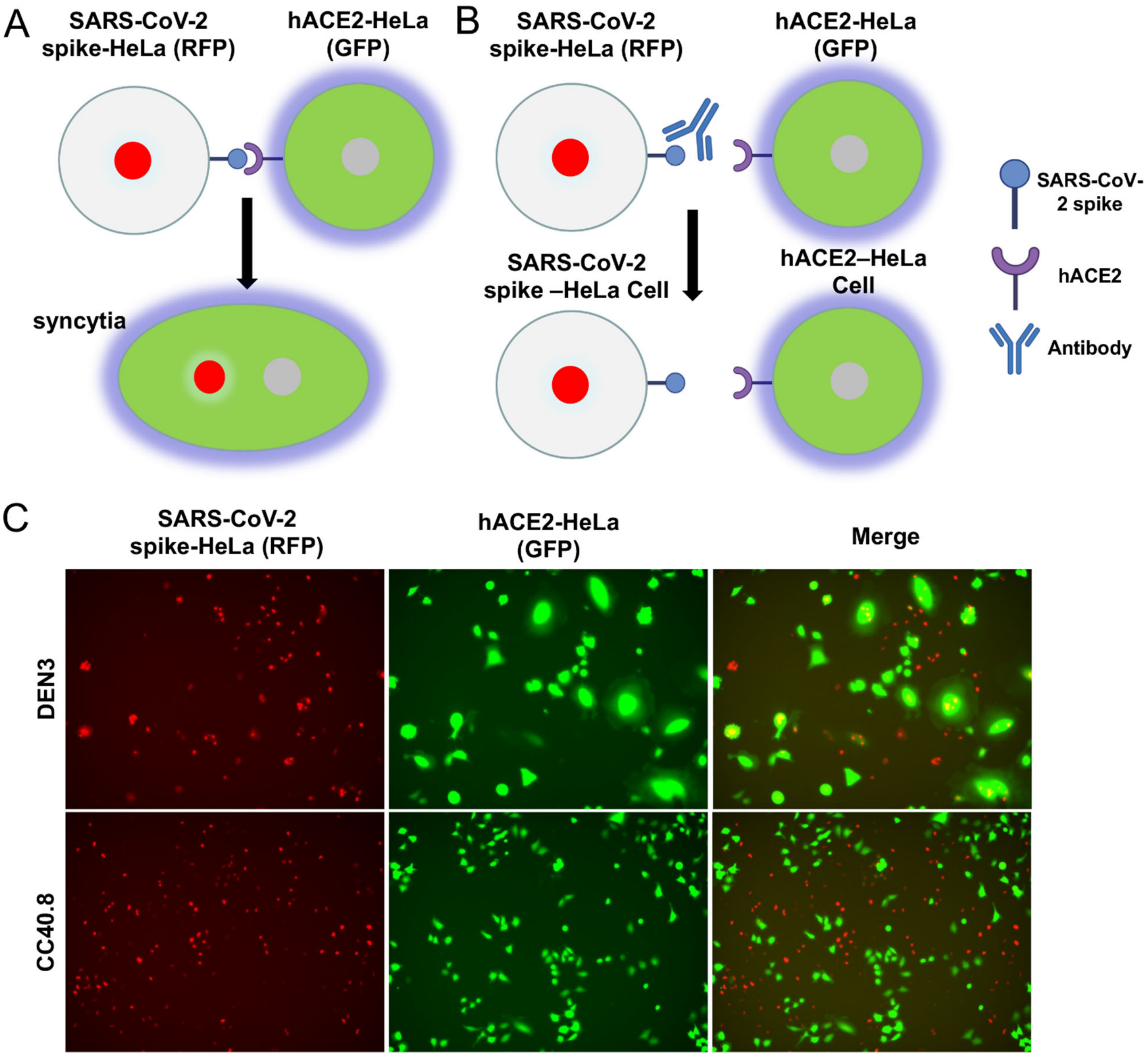

Fig. S2. CC40.8 antibody inhibits SARS-CoV-2 spike protein- and hACE2-mediated cell-cell fusion.

(A and B) A schematic diagram of cell-cell fusion assay is shown. SARS-CoV-2 spike-HeLa cells express nucleus-restricted RFP (Red) and hACE2-HeLa cells express cytosolic GFP (Green). The interaction of SARS-CoV-2 spike protein and hACE2 can lead to cell fusion to form syncytia. In the same syncytium, both GFP in the cytoplasm and RFP in the nucleus can be seen (A). If antibody can block cell-cell fusion, no syncytia can be seen. Only GFP-expressing hACE2-HeLa cells and RFP-expressing SARS-CoV-2 spike-HeLa cells can be seen (B).

(C) SARS-CoV-2 spike-HeLa cells (red) were pre-incubated with negative control antibody (DEN3) or CC40.8 S2 stem bnAb for 1 hour, and then mixed with hACE2-HeLa cells (green). Green syncytia were observed with DEN3, indicating widespread cell-cell fusion mediated by SARS-CoV-2 spike and hACE2; fusion was inhibited by addition of CC40.8.

Peptide number	HKU1 S2 residues	Sequence	OD405nm	
			CC40.8	DEN3
1	761-775	SISASYRFVTFEPFN	0.2	0.2
2	766-780	YRFVTFEPFNVSFVN	0.2	0.2
3	771-785	FEPFNVSFVNDSIES	0.1	0.2
4	776-790	VSFVNDSIESVGGLY	0.1	0.2
5	781-795	DSIESVGGLYEIKIP	0.2	0.2
6	786-800	VGGLYEIKIPTNFTI	0.1	0.2
7	791-805	EIKIPTNFTIVGQEE	0.1	0.2
8	796-810	TNFTIVGQEEFIQTN	0.1	0.2
9	801-815	VGQEEFIQTNSPKVT	0.1	0.2
10	806-820	FIQTNSPKVTIDCSL	0.2	0.2
11	811-825	SPKVTIDCSLFVCSN	0.2	0.2
12	816-830	IDCSLFVCSNYAACH	0.2	0.2
13	821-835	FVCSNYAACHDLLSE	0.2	0.3
14	826-840	YAACHDLLSEYGTFC	0.2	0.3
15	831-845	DLLSEYGTFCDNINS	0.2	0.3
16	836-850	YGTFCDNINSILDEV	0.2	0.2
17	841-855	DNINSILDEVNGLLD	0.1	0.2
18	846-860	ILDEVNGLLDTTQLH	0.1	0.2
19	851-865	NGLLDTTQLHVADTL	0.1	0.2
20	856-870	TTQLHVADTLMQGVT	0.1	0.2
21	861-875	VADTLMQGVTLSSNL	0.1	0.2
22	866-880	MQGVTLSSNLNTNLH	0.1	0.2
23	871-885	LSSNLNTNLHFDVDN	0.1	0.2
24	876-890	NTNLHFDVDNINFKS	0.1	0.2
25	881-895	FDVDNINFKSLVGCL	0.2	0.3
26	886-900	INFKSLVGCLGPHCG	0.2	0.3
27	891-905	LVGCLGPHCGSSSRS	0.2	0.2
28	896-910	GPHCGSSSRSFFEDL	0.2	0.2
29	901-915	SSSRSFFEDLLFDKV	0.2	0.2
30	906-920	FFEDLLFDKVKLSDV	0.1	0.2
31	911-925	LFDKVKLSDVGFVEA	0.2	0.2
32	916-930	KLSDVGFVEAYNNCT	0.2	0.3
33	921-935	GFVEAYNNCTGGSEI	0.2	0.2
34	926-940	YNNCTGGSEIRDLLC	0.2	0.2
35	931-945	GGSEIRDLLCVQSFN	0.2	0.2
36	936-950	RDLLCVQSFNGIKVL	0.2	0.2
37	941-955	VQSFNGIKVLPPILS	0.2	0.2
38	946-960	GIKVLPPILSESQIS	0.2	0.2
39	951-965	PPILSESQISGYTTA	0.2	0.2
40	956-970	ESQISGYTTAATVAA	0.1	0.2
41	961-975	GYTTAATVAAMFPPW	0.2	0.2
42	966-980	ATVAAMFPPWSAAAG	0.1	0.2
43	971-985	MFPPWSAAAGIPFSL	0.2	0.2
44	976-990	SAAAGIPFSLNVQYR	0.2	0.2
45	981-995	IPFSLNVQYRINGLG	0.2	0.2
46	986-1000	NVQYRINGLGVTMDV	0.2	0.2
47	991-1005	INGLGVTMDVLNKNQ	0.1	0.2
48	996-1010	VTMDVLNKNQKLIAT	0.2	0.2
49	1001-1015	LNKNQKLIATAFNNA	0.2	0.2
50	1006-1020	KLIATAFNNALLSIQ	0.2	0.2
51	1011-1025	AFNNALLSIQNGFSA	0.2	0.2
52	1016-1030	LLSIQNGFSATNSAL	0.1	0.2
53	1021-1035	NGFSATNSALAKIQS	0.2	0.2
54	1026-1040	TNSALAKIQSVVNSN	0.2	0.2
55	1031-1045	AKIQSVVNSNAQALN	0.2	0.2
56	1036-1050	VVNSNAQALNSLLQQ	0.2	0.2
57	1041-1055	AQALNSLLQQLFNKF	0.2	0.3
58	1046-1060	SLLQQLFNKFGAISS	0.2	0.2
59	1051-1065	LFNKFGAISSSLQEI	0.2	0.2
60	1056-1070	GAISSSLQEILSRLD	0.2	0.2
61	1061-1075	SLQEILSRLDALEAQ	0.2	0.3
62	1066-1080	LSRLDALEAQVQIDR	0.2	0.2
63	1071-1085	ALEAQVQIDRLINGR	0.2	0.2
64	1076-1090	VQIDRLINGRLTALN	0.2	0.2
65	1081-1095	LINGRLTALNAYVSQ	0.2	0.2
66	1086-1100	LTALNAYVSQQLSDI	0.2	0.3
67	1091-1105	AYVSQQLSDISLVKF	0.2	0.3
68	1096-1110	QLSDISLVKFGAALA	0.2	0.2
69	1101-1115	SLVKFGAALAMEKVN	0.2	0.2
70	1106-1120	GAALAMEKVNECVKS	0.2	0.2
71	1111-1125	MEKVNECVKSQSPRI	0.2	0.3
72	1116-1130	ECVKSQSPRINFCGN	0.2	0.2
73	1121-1135	QSPRINFCGNGNHIL	0.2	0.2
74	1126-1140	NFCGNGNHILSLVQN	0.2	0.2
75	1131-1145	GNHILSLVQNAPYGL	0.2	0.2
76	1136-1150	SLVQNAPYGLLFMHF	0.2	0.3
77	1141-1155	APYGLLFMHFSYKPI	0.2	0.3
78	1146-1160	LFMHFSYKPISFKTV	0.2	0.2
79	1151-1165	SYKPISFKTVLVSPG	0.2	0.2
80	1156-1170	SFKTVLVSPGLCISG	0.2	0.3
81	1161-1175	LVSPGLCISGDVGIA	0.2	0.2
82	1166-1180	LCISGDVGIAPKQGY	0.2	0.2
83	1171-1185	DVGIAPKQGYFIKHN	0.2	0.2
84	1176-1190	PKQGYFIKHNDHWMF	0.2	0.2
85	1181-1195	FIKHNDHWMFTGSSY	0.2	0.3
86	1186-1200	DHWMFTGSSYYYPEP	0.2	0.2
87	1191-1205	TGSSYYYPEPISDKN	0.2	0.2
88	1196-1210	YYPEPISDKNVVFMN	0.2	0.2
89	1201-1215	ISDKNVVFMNTCSVN	0.2	0.2
90	1206-1220	VVFMNTCSVNFTKAP	0.2	0.2
91	1211-1225	TCSVNFTKAPLVYLN	0.2	0.3
92	1216-1230	FTKAPLVYLNHSVPK	0.2	0.2
93	1221-1235	LVYLNHSVPKLSDFE	0.2	0.2
94	1226-1240	HSVPKLSDFESELSH	0.2	0.2
95	1231-1245	LSDFESELSHWFKNQ	2.5	0.2
96	1236-1250	SELSHWFKNQTSIAP	0.2	0.2
97	1241-1255	WFKNQTSIAPNLTLN	0.2	0.2
98	1246-1260	TSIAPNLTLNLHTIN	0.2	0.2
99	1251-1265	NLTLNLHTINATFLD	0.2	0.2
100	1256-1270	LHTINATFLDLYYEM	0.2	0.2
101	1261-1275	ATFLDLYYEMNLIQE	0.2	0.2
102	1266-1280	LYYEMNLIQESIKSL	0.2	0.2
103	1271-1285	NLIQESIKSLNNSYI	0.2	0.2
104	1276-1290	SIKSLNNSYINLKDI	0.2	0.2
105	1281-1295	NNSYINLKDIGTYEM	0.2	0.2
106	1286-1300	NLKDIGTYEMYVKWP	0.2	0.2
107	1291-1305	GTYEMYVKWPWYVWL	0.3	0.3
108	1296-1310	YVKWPWYVWLLISFS	0.3	0.2
109	1301-1315	WYVWLLISFSFIIFL	0.2	0.2
110	1306-1320	LISFSFIIFLVLLFF	0.1	0.2
111	1311-1325	FIIFLVLLFFICCCT	0.2	0.2
112	1316-1330	VLLFFICCCTGCGSA	0.2	0.3
113	1321-1335	ICCCTGCGSACFSKC	0.2	0.3
114	1326-1340	GCGSACFSKCHNCCD	0.2	0.3
115	1331-1345	CFSKCHNCCDEYGGH	0.2	0.2
116	1336-1350	HNCCDEYGGHHDFVI	0.2	0.3
117	1341-1355	EYGGHHDFVIKTSHD	0.2	0.2

Fig. S3. Epitope mapping of CC40.8 antibody with HCoV-HKU1 S2 subunit-derived overlapping peptides.

ELISA binding results are shown for CC40.8 mAb with HCoV-HKU1 S2 subunit overlapping peptides (residue number range: 761-1355). Each HCoV-HKU1 S2 subunit peptide is 15-residues long with a 10-residue overlap. Peptide IDs, S2 subunit residue number ranges of 15-mer peptides, and antibody binding responses are shown. CC40.8 exhibited binding to the 95th peptide (residue position range: 1231-1245) corresponding to the HCoV-HKU1 S2 stem-helix region. DEN3, an antibody to dengue virus, was used as a control.

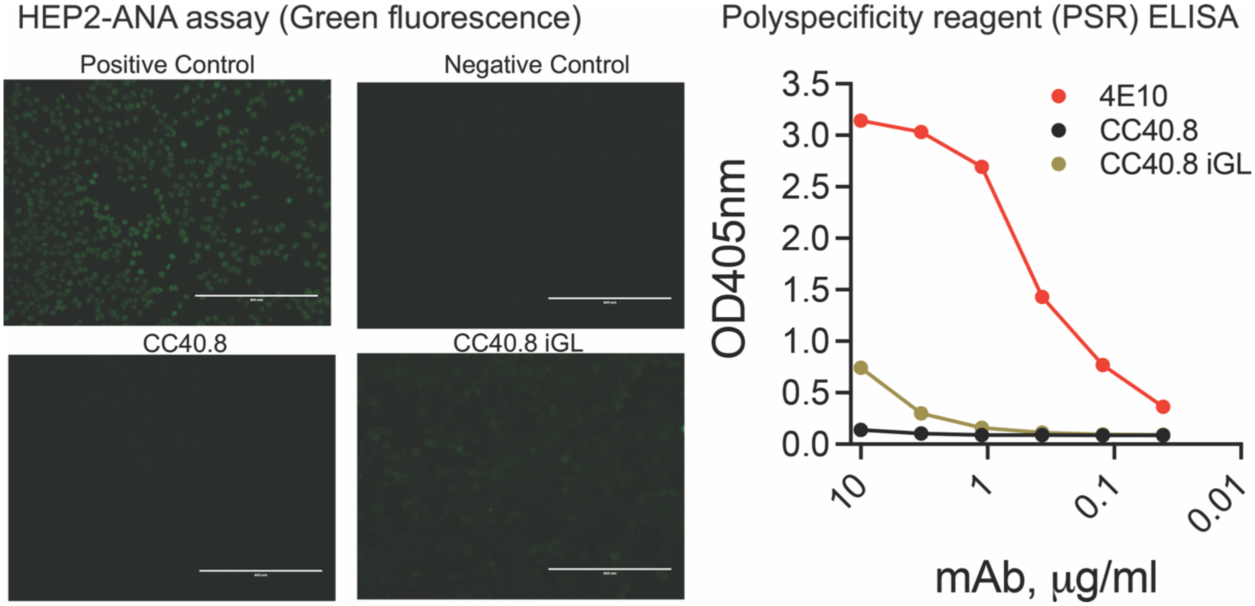

Fig. S4. Polyreactivity analysis of CC40.8 and CC40.8 iGL antibodies.
(Left) Immunofluorescence showing binding of antibodies to immobilized HEp2 epithelial cells was detected by FITC-labelled secondary antibody. Positive and negative controls for the Hep2 kit assay are provided by the manufacturer. (Right) Antibodies were tested by enzyme-linked immunosorbent assay (ELISA) for binding to the polyspecificity reagent (PSR) from CHO-cell solubilized membrane protein (SMP). 4E10, an HIV MPER-specific antibody known to display polyreactivity, was used as a positive control.

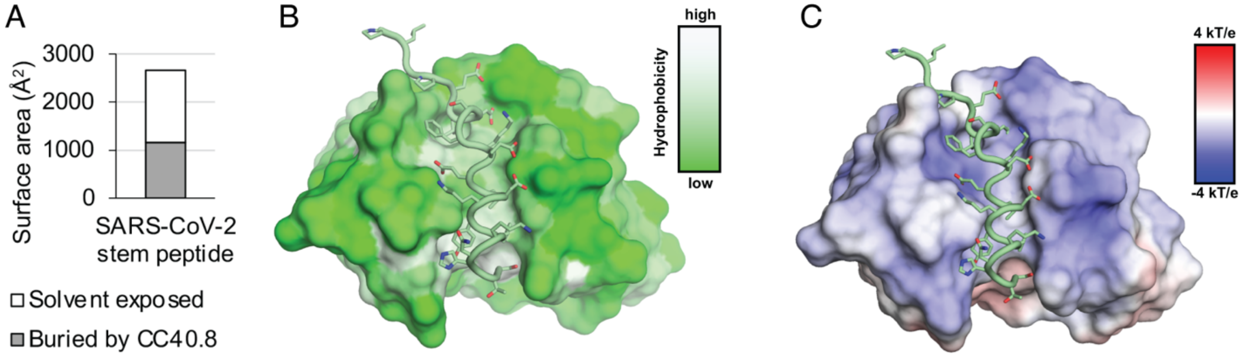

Fig. S5. CC40.8 bnAb structure with SARS-CoV-2 S2 stem-helix peptide.

(A) Surface area of the SARS-CoV-2 stem peptide is shown. Solvent exposed and buried areas were calculated with Proteins, Interfaces, Structures and Assemblies (PISA) (*91*).

(B) The SARS-CoV-2 stem peptide inserts into a hydrophobic groove formed by the heavy and light chains of CC40.8. Surfaces of CC40.8 are color-coded by hydrophobicity [calculated by Color h (https://pymolwiki.org/index.php/Color_h)].

(C) Electrostatic surface potential of the CC40.8 paratope is shown. Electrostatic potential was calculated by APBS and PDB2PQR (*93, 94*).

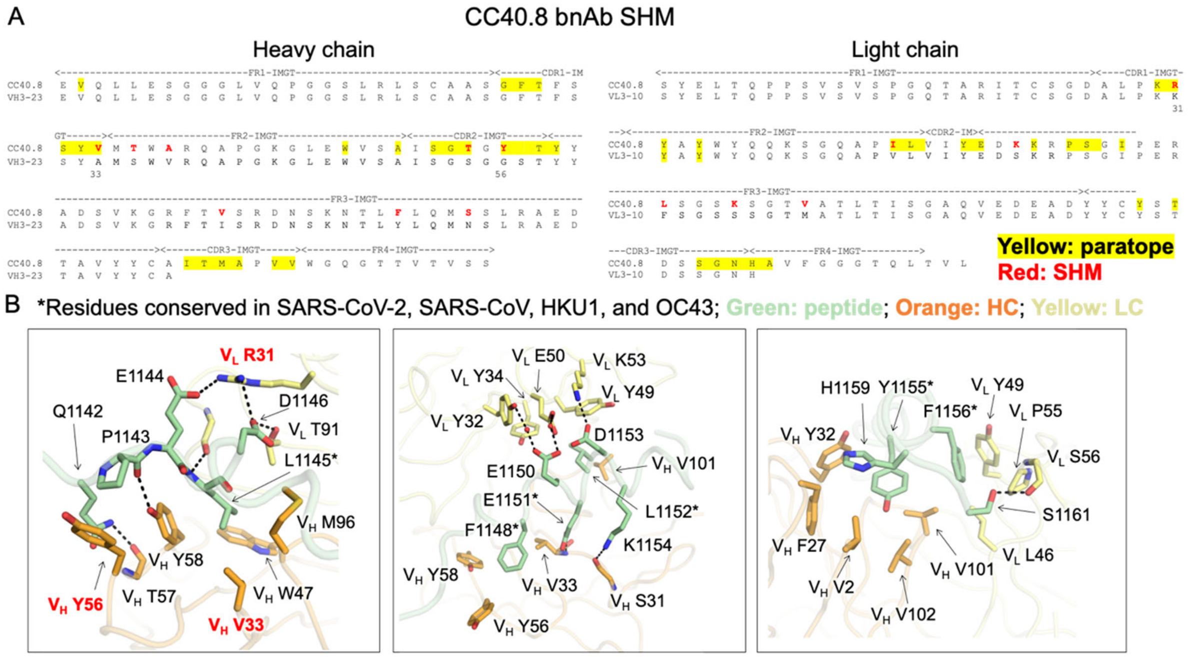

Fig. S6. Contribution of CC40.8 bnAb heavy and light chain germline and somatic mutated V-gene residues in S2 stem epitope recognition.

(A) Alignment of CC40.8 with germline VH3-23 and VL3-10 sequences is shown. Paratope residues [defined as buried surface area (BSA) > 0 Å^2 as calculated by PISA (*91*)] are highlighted with yellow boxes. Somatically mutated residues as calculated by IgBLAST (*95*) are highlighted in red.

(B) Detailed interactions between CC40.8 Fab and the SARS-CoV-2 stem peptide are shown. Heavy and light chains of CC40.8 are shown in orange and yellow, and the SARS-CoV-2 stem peptide is in pale green. Hydrogen bonds and salt bridges are represented by black dashed lines. Somatically mutated residues are shown in red. Conserved residues among coronaviruses are indicated by asterisks (*).

Alanine scanning of SARS-CoV-2 Spike

	SARS-CoV-2	WT	P1140A	L1141A	Q1142A	P1143A	E1144A	L1145A	D1146A	S1147A	F1148A	K1149A	E1150A	E1151
Neutralization	IC50 (ug/ml, CC40.8)	11.5	1.4	0.6	3.3	238.4	14.6	N/A	84.0	20.5	>300	0.6	24.2	>300
	n-fold	1.0	0.1	0.1	0.3	20.7	1.3	N/A	7.3	1.8	>26.11	0.0	2.1	>26.11
BLI Binding	Response Value (CC40.8)	0.61	0.57	N/A	0.59	0.21	0.62	0.02	0.52	0.55	0.35	0.61	0.51	0.07
	% change with WT	100%	95%	N/A	97%	35%	102%	4%	86%	90%	58%	101%	83%	12%
	Response Value (S309)	0.43	0.43	N/A	0.41	0.45	0.42	0.40	0.43	0.45	0.44	0.42	0.48	0.47
	% change with WT	100%	100%	N/A	94%	103%	97%	91%	100%	103%	101%	98%	110%	108%
	SARS-CoV-2	L1152A	D1153A	K1154A	Y1155A	F1156A	K1157A	N1158A	H1159A	T1160A	S1161A	P1162A	D1163A	V1164A
Neutralization	IC50 (ug/ml, CC40.8)	143.1	71.0	4.4	>300	>300	4.7	4.5	6.7	1.9	6.4	11.6	16.0	6.6
	n-fold	12.5	6.2	0.4	>26.11	>26.11	0.4	0.4	0.6	0.2	0.6	1.0	1.4	0.6
BLI Binding	Response Value (CC40.8)	0.65	0.62	0.74	0.34	0.64	0.74	0.65	0.69	0.65	0.49	0.52	0.60	0.75
	% change with WT	108%	102%	122%	55%	106%	122%	107%	114%	107%	81%	86%	99%	124%
	Response Value (S309)	0.48	0.44	0.42	0.46	0.49	0.50	0.43	0.51	0.55	0.47	0.40	0.45	0.47
	% change with WT	110%	101%	98%	107%	113%	114%	99%	117%	127%	108%	93%	103%	110%

Neutralization
n-fold <0.3
n-fold >5

BLI Binding
% change <80%

Alanine scanning of SARS-CoV-1/2 S2 stem peptide

	SARS-CoV-1/2	WT	P1140A	L1141A	Q1142A	P1143A	E1144A	L1145A	D1146A	S1147A	F1148A	K1149A	E1150A	E1151
BLI Binding	Response Value (CC40.8)	3.8	3.5	3.8	3.8	3.7	3.5	0.9	3.3	3.3	2.3	3.7	3.5	0.6
	% change with WT	100%	92%	99%	99%	97%	92%	22%	86%	87%	61%	96%	91%	16%
	SARS-CoV-1/2	L1152A	D1153A	K1154A	Y1155A	F1156A	K1157A	N1158A	H1159A	T1160A	S1161A	P1162A	D1163A	V1164A
BLI Binding	Response Value (CC40.8)	2.9	3.0	3.4	2.1	2.4	3.7	3.0	2.6	3.2	3.3	3.2	2.8	2.5
	% change with WT	76%	80%	89%	54%	62%	97%	79%	68%	84%	87%	83%	74%	66%

Alanine scanning of HCoV-HKU1 S2 stem peptide

	HCoV-HKU1	WT	H1140A	S1141A	V1142A	P1143A	K1144A	L1145A	S1146A	D1147A	F1148A	E1149A	S1150A	E1151A
BLI Binding	Response Value (CC40.8)	4.2	4.6	4.3	4.4	4.4	4.4	3.3	4.4	4.0	4.0	3.6	4.0	1.7
	% change with WT	100%	110%	104%	105%	106%	105%	79%	105%	96%	96%	87%	96%	41%
	HCoV-HKU1	L1152A	S1153A	H1154A	W1155A	K1156A	F1157A	N1158A	Q1159A	T1160A	S1161A	I1162A	A1163A	P1164A
BLI Binding	Response Value (CC40.8)	3.7	4.0	3.8	1.8	2.7	4.5	4.2	4.2	4.3	4.3	4.3	4.2	3.8
	% change with WT	88%	96%	91%	43%	64%	107%	101%	100%	104%	104%	104%	100%	91%

Fig. S7. Epitope mapping of CC40.8 bnAb by alanine scanning mutagenesis of SARS-CoV-2 spike protein and SARS-CoV-2/HCoV-HKU1 S2 stem peptides using neutralization and BLI binding assays.

The upper panel shows the IC_{50} neutralization of CC40.8 bnAb with wild-type (WT) SARS-CoV-2 and spike mutant pseudoviruses and the BLI binding responses with WT SARS-CoV-2 soluble spike protein and alanine mutants. SARS-CoV-2 receptor binding domain (RBD) antibody S309 was a control for the spike protein binding assays. The IC_{50} fold change (n-fold) was calculated by dividing the mutant value by the WT value. For IC_{50}, n-fold <0.3 are indicated in red, and n-fold >5 in orange. The middle and lower panels show BLI binding responses of CC40.8 antibody to WT and alanine mutants of the SARS-CoV-1/2 and HCoV-HKU1 stem-helix peptides, respectively. Binding response values where the % change in binding (from WT peptide) is below 80% are indicated in yellow. Antibody S309 that recognizes a fairly conserved epitope of the RBD of both SARS-CoV-1 and SARS-CoV-2 was used as control. N/A, not available.

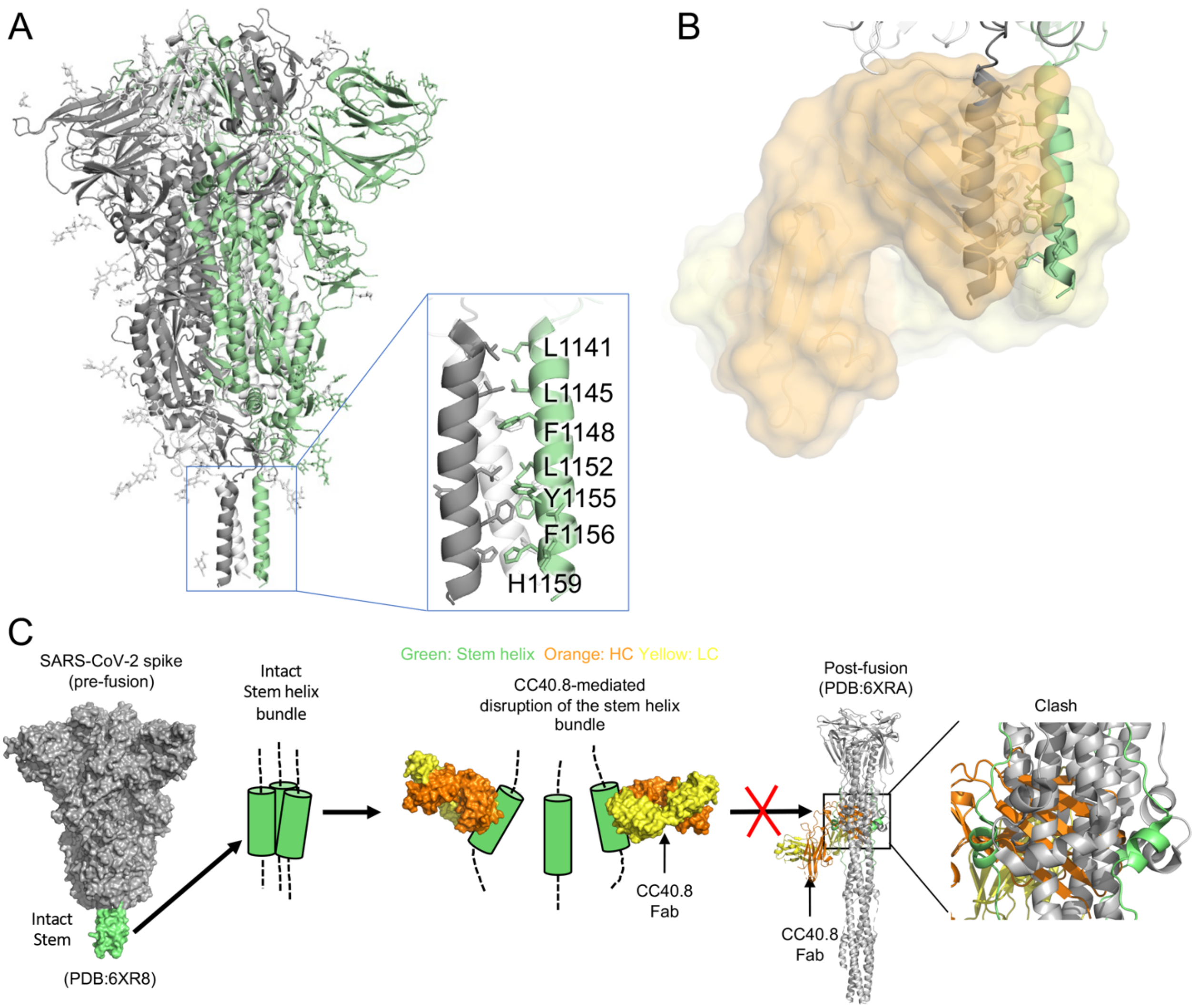

Fig. S8. CC40.8 binds to a buried interface of the 3-helix bundle: predicted mechanism of neutralization.

(A) A SARS-CoV-2 spike protein structure is shown in the pre-fusion state. The three protomers are shown in gray, pale green, and white, respectively, with N-linked glycans represented by sticks. The 3-helix bundle stem region is highlighted in a blue-outlined box. Representative epitope residues of CC40.8 are shown in sticks. The CC40.8 epitope is rich in hydrophobic residues. A cryo-EM structure of SARS-CoV-2 spike protein structure in the pre-fusion state that incorporates the coordinates of the 3-helix bundle stem region (PDB: 6XR8, (*96*)) is shown here.

(B) The SARS-CoV-2 spike protein pre-fusion structure was superimposed on the structure of CC40.8 (orange/yellow) in complex with a SARS-CoV-2 S2 peptide. CC40.8 would clash with the other protomers of the spike protein in the pre-fusion state.

(C) A putative neutralization mechanism of CC40.8 is presented. The S2 3-helix bundle region is shown in green, and heavy and light chains of CC40.8 are shown in orange and yellow, respectively. A model for the mechanism of neutralization is shown and inspired by the interaction of a mouse S2 stem antibody, B6, isolated from a spike protein vaccinated animal that targets a similar stem epitope (*52*).

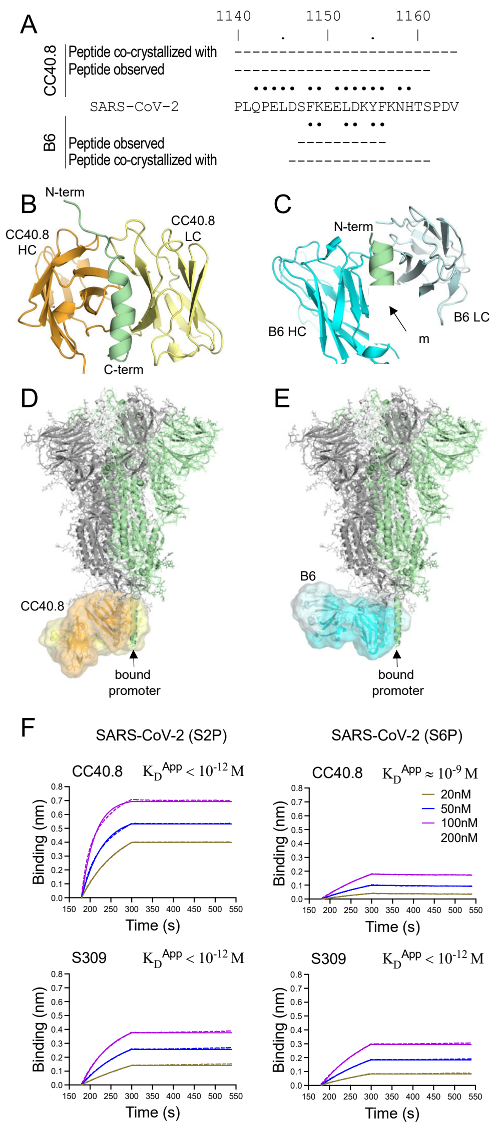

Fig. S9. Comparison of bnAbs CC40.8 and B6 that target the S2 stem helix.
(A) A comparison between S2 stem-helix peptides targeted by CC40.8 and B6 is shown. Peptides used for co-crystallization with CC40.8 or B6 are indicated by dashes, with the regions observed in the crystal structures of each study indicated. Residues involved in interactions with CC40.8 and B6 are indicated by black dots (cutoff distance = 4 Å).
(B to E) Structures of CC40.8 (B) and B6 (C) were compared. The heavy and light chains of CC40.8 are colored in orange and yellow, respectively, and those for B6 are in cyan and light cyan. The S2 stem-helix peptides are shown in green. In panels (D) and (E), a SARS-CoV-2 spike protein pre-fusion structure (PDB 6XR8) is superimposed on structures of CC40.8 and B6 in complex with a SARS-CoV-2 S2 peptide in the green protomer (indicated by arrows). Both CC40.8 and B6 would clash with the other protomers of the spike protein in pre-fusion state.
(F) BLI binding kinetics of CC40.8 to S2- and S6- stabilized SARS-CoV-2 spike trimers are shown. An RBD-targeting neutralizing Ab S309 (*97*) was used as a control. Apparent binding constants (K_D^{App}) of antibodies with spike proteins are shown. The raw experimental curves are shown as dashed lines, and the solid lines are the fits.

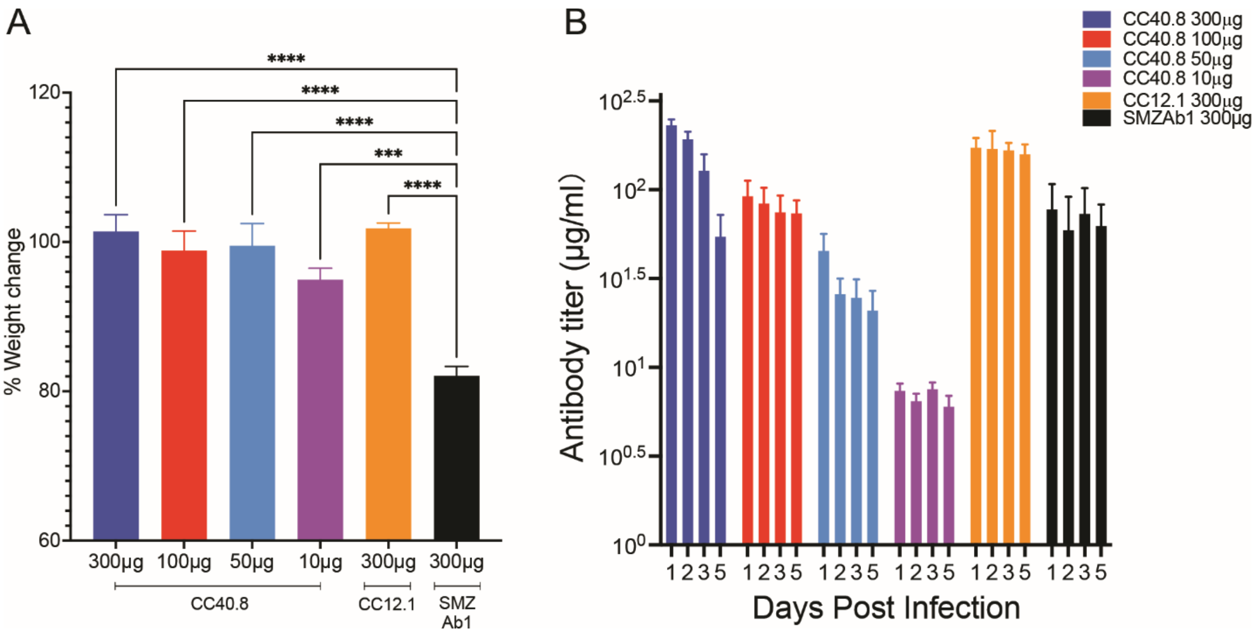

Fig. S10. Weight loss, viral titers, and serum antibody titers were measured in hACE2 mice passively administered CC40.8.

(A) Percent day 5 weight change was calculated from day 0 for all animals. Data are presented as mean ± SEM. Significance was calculated with Dunnett's multiple comparisons test between each experimental group and the Zika virus monoclonal Ab (SMZAb1) control group (***P<0.001, ****P<0.0001).

(B) Serum human IgG concentrations of CC40.8, CC12.1 and SMZAb1 were assessed by ELISA at day 1, 2, 3, and 5 post infection. Data are presented as mean ± SEM.

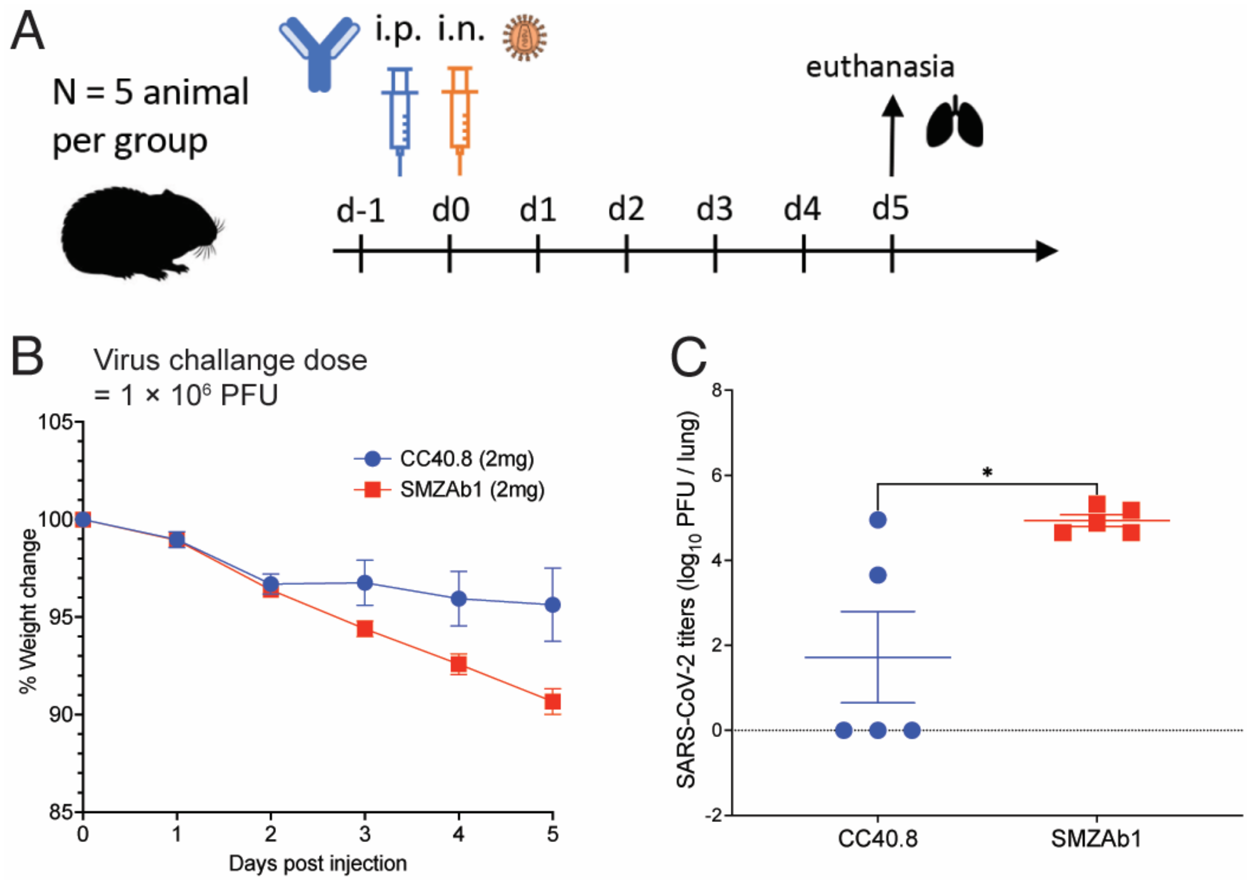

Fig. S11. CC40.8 reduces weight loss and lung viral load and viral replication following SARS-CoV-2 challenge in Syrian hamsters.

(A) CC40.8 was administered intraperitonially (i.p.) at a 2 mg per animal dose into Syrian hamsters (average: 16.5 mg/kg). Control animals received 2 mg of control SMZAb1. Each group of five animals was challenged intranasally (i.n.) 12 hours after antibody infusion with 1 × 10^6 plaque-forming units (PFU) of SARS-CoV-2. Animal weight was monitored daily as an indicator of disease progression and lung tissue was collected on day 5 for viral burden assessment.

(B) Percent weight change is shown for CC40.8 or control antibody-treated animals after SARS-CoV-2 challenge. Percent weight change was calculated from day 0 for all animals. Data are presented as mean ± SEM.

(C) SARS-CoV-2 titers (PFU) were determined by plaque assay from lung tissue at day 5 after infection. Three out of 5 CC40.8-treated animals had substantially reduced viral titers compared to the SMZAb1 control antibody-treated animals. Data are presented as mean ± SEM.

Table S1. X-ray data collection and refinement statistics.

**Data collection**	CC40.8 Fab + SARS-CoV-2 S2 peptide
Beamline	SSRL12-1
Wavelength (Å)	0.97946 Å
Space group	$P\ 2\ 2_1\ 2_1$
Unit cell parameters	
a, b, c (Å)	54.9, 63.7, 122.0
α, β, γ (°)	90, 90, 90
Resolution (Å) [a]	50.0-1.62 (1.65-1.62)
Unique reflections [a]	54,176 (4,897)
Redundancy [a]	4.3 (3.0)
Completeness (%) [a]	97.0 (89.6)
$<I/\sigma_I>$ [a]	29.9 (1.0)
R_{sym}[b] (%) [a]	7.9 (>100)
R_{pim}[b] (%) [a]	2.8 (47.7)
$CC_{1/2}$[c] (%) [a]	99.4 (56.3)
**Refinement statistics**	
Resolution (Å)	29.1-1.62
Reflections (work)	54,129
Reflections (test)	1,997
R_{cryst}[d] / R_{free}[e] (%)	17.4/20.6
No. of atoms	3,836
Fab	3,159
Peptide	193
Ligands	35
Solvent	459
Average B-values (Å^2)	28
Fab	26
Peptide	34
Ligands	56
Solvent	39
Wilson B-value (Å^2)	23
**RMSD from ideal geometry**	
Bond length (Å)	0.006
Bond angle (°)	1.22
**Ramachandran statistics (%)** [g]	
Favored	98.2
Outliers	0.0
**PDB code**	7SJS

[a] Numbers in parentheses refer to the highest resolution shell.
[b] $R_{sym} = \Sigma_{hkl} \Sigma_i | I_{hkl,i} - <I_{hkl}> | / \Sigma_{hkl} \Sigma_i I_{hkl,i}$ and $R_{pim} = \Sigma_{hkl} (1/(n-1))^{1/2} \Sigma_i | I_{hkl,i} - <I_{hkl}> | / \Sigma_{hkl} \Sigma_i I_{hkl,i}$, where $I_{hkl,i}$ is the scaled intensity of the ith measurement of reflection h, k, l, $<I_{hkl}>$ is the average intensity for that reflection, and n is the redundancy.
[c] $CC_{1/2}$ = Pearson correlation coefficient between two random half datasets.
[d] $R_{cryst} = \Sigma_{hkl} | F_o - F_c | / \Sigma_{hkl} | F_o |$ x 100, where F_o and F_c are the observed and calculated structure factors, respectively.
[e] R_{free} was calculated as for R_{cryst}, but on a test set comprising 5% of the data excluded from refinement.
[g] From MolProbity (*98*).

Table S2. Demographic information of COVID-19 convalescent donors.

	**COVID donor (n = 60)**
**Age** (years)	20 to 72 (median = 46)
**Gender**	
Male	47% (28/60)
Female	53% (32/60)
**Race/Ethnicity**	
White, non-Hispanic	80% (48/60)
Hispanic	8.3% (5/60)
Black, non-Hispanic	1.7% (1/60)
Asian, non-Hispanic	3.3% (2/60)
Unknown	6.7% (4/60)
SARS-CoV-2 PCR Positivity	75% (45/60)
Lateral Flow Positivity	60% (36/60)
**Disease Severity**	
Mild	56.7% (34/60)
Mild to Moderate	6.7% (4/60)
Moderate	25% (15/60)
Moderate to Severe	5% (3/60)
Severe	5% (3/60)
Critical	1.7% (1/60)
**Symptoms**	
Cough	60% (36/60)
Fever	55% (33/60)
Fatigue	38.3% (23/60)
Anosmia	31.7% (19/60)
Dyspnea	26.7% (16/60)
Diarrhea	16.7% (10/60)
Days Post Symptom Onset at Collection	6 to 90 (median = 35.5)
